# Mis-localized nucleoporin POM121C potently inhibits nuclear entry of HIV-1 but leads to the outgrowth of viral escape mutants

**DOI:** 10.1128/jvi.00312-26

**Published:** 2026-07-23

**Authors:** Hideki Saito, Eri Miyagi, Sandra Kao, Annie Dai, Mohammad M. Rashid, Darian T. Yang, Rajasree Chakraborty, Chandravanu Dash, Angela M. Gronenborn, Felipe Diaz-Griffero, Klaus Strebel

**Affiliations:** 1 Viral Biochemistry Section, Laboratory of Molecular Microbiology, National Institute of Allergy and Infectious Diseases, National Institutes of Health 2511, Bethesda, Maryland, USA; 2 Department of Microbiology and Immunology, Albert Einstein College of Medicine 2006, Bronx, New York, USA; 3 Department of Structural Biology, School of Medicine, University of Pittsburgh 6614, Pittsburgh, Pennsylvania, USA; 4 Molecular Biophysics and Structural Biology Graduate Program, University of Pittsburgh and Carnegie Mellon University 6614, Pittsburgh, Pennsylvania, USA; 5 Department of Microbiology, Immunology, and Physiology, School of Medicine, The Center for AIDS Health Disparities Research, Meharry Medical College 5708, Nashville, Tennessee, USA; University Hospital Tübingen, Tübingen, Germany

**Keywords:** human immunodeficiency virus, virus–host interactions, restriction factor, nuclear import, escape mutants, POM121C, POM121

## Abstract

Nuclear entry of HIV-1 involves specific interactions between the viral capsid and components of the nuclear pore complex. FG-repeat sequences present in many nucleoporins play important roles in HIV-1 nuclear entry. We previously reported that overexpression of the FG repeat-containing C-terminal fragment of the nucleoporin POM121C (residues 614–987), termed POM987, inhibits HIV-1 infection. However, the mechanism underlying this inhibition remained unclear. Here, we identified HIV-1 escape mutants that are resistant to POM987-mediated restriction and mapped the responsible mutations to two amino acid positions in the capsid protein. POM987 bound the incoming HIV-1 capsid core and inhibited viral nuclear entry. In contrast, the escape mutant showed reduced interaction with POM987 and restored nuclear entry and integration in POM987-expressing cells. These findings indicate that the antiviral activity of POM987 is mediated by capsid-dependent inhibition of HIV-1 nuclear entry and suggest that capsid–nucleoporin interactions could provide a potential target for developing novel antiviral therapies.

**IMPORTANCE:**

HIV-1 can infect non-dividing cells. This requires interaction with nuclear pore proteins, including POM121C. A mis-localized fragment of POM121C potently inhibits nuclear entry through direct interaction with the incoming capsid core. Escape variants acquire mutations at specific capsid residues, enabling them to evade this interaction and thereby restore nuclear import.

## INTRODUCTION

Nuclear import of HIV-1 is a critical step in the virus replication cycle, enabling the viral genome to integrate into the host DNA. A unique feature of HIV-1 is the ability to infect non-dividing cells, such as macrophages. This process involves the coordinated interaction of viral and host cell factors (1–4). Specifically, the HIV-1 capsid (CA) core plays a central role in nuclear import (5–7). The HIV-1 CA interacts with components of the nuclear pore complex (NPC) such as Nup358, Nup153, and Nup98, thereby hijacking specific nuclear transport pathways (7–12). CPSF6 and TNPO3 also associate with CA to facilitate transport of the pre-integration complex (PIC) through the nuclear pore (13–17). However, whether HIV-1 requires nuclear transport factors such as KPNB1, KPNA2, and importin 7 remains unclear, as conflicting reports exist (18–21). Despite the established importance of CA–host factor interactions, the precise mechanism by which the HIV-1 core transits the nuclear pore remains unresolved. Intact HIV-1 capsids, estimated to be 55 nm or larger (22, 23), exceed the estimated diameter of the nuclear pore channel, raising the question of how the viral core crosses the nuclear envelope. Earlier models proposed that the capsid disassembles before nuclear import to form a PIC containing viral proteins such as MA, NC, RT, IN, and Vpr (24, 25). However, recent imaging studies have suggested that largely intact capsid cores can dock within or traverse the nuclear pore, whereas other studies indicate that CA loss at the nuclear pore precedes productive infection (26–28). Thus, HIV-1 nuclear import likely involves dynamic capsid remodeling at the nuclear pore, but the molecular details of this process remain to be defined.

The timing and location of reverse transcription in relation to nuclear import also remain under debate, with models suggesting its completion in the cytoplasm, at the nuclear pore, or even within the nucleus (4, 29–34). Despite the complexity of HIV-1 nuclear import, recent therapeutic advances have targeted this process. For instance, the FDA-approved capsid inhibitor Lenacapavir potently inhibits an early stage of HIV-1 replication by competitively blocking interactions between CA and host factors such as NUP153 and CPSF6 (35–38). Given the crucial role of the HIV-1 core in viral replication and antiviral treatment, it is not surprising that mutations affecting CA structure and function influence replication fitness and immune evasion. For example, mutations in CA such as P38A, E45A, K203A, or Q219A alter capsid stability and impact reverse transcription and nuclear import (39–41). Other mutations, such as N74D (disrupting TNPO3 and CPSF6 binding) (14, 16) or P90A (which alters Nup153 and cyclophilin A interaction) (42), affect host factor interactions, influence nuclear import, and alter cell-type specificity of viral infection.

POM121C is a paralog of POM121, which we refer to as POM121A in this study. Both genes are located on chromosome 7 (43) and share structural and functional similarities. Both are integral nuclear membrane proteins and presumably have a role in anchoring the NPCs in the nuclear membrane (44). However, their precise physiological roles remain unclear (43, 45).

We previously reported that ectopic overexpression of a truncated form of the FG repeat-containing C-terminal region of POM121C (referred to in the following as POM987) strongly inhibited HIV-1 infection, possibly interfering with the nuclear import step (46). Subsequent studies established a role for POM121A in HIV-1 infection by showing that knockdown of endogenous POM121A using siRNA inhibits viral infection. Indeed, there is now evidence that Nup35, Nup153, and POM121A cooperatively facilitate HIV-1 nuclear entry (47, 48). However, the detailed mechanism was unclear.

Here, we investigated the mechanism of POM121C-mediated inhibition of HIV-1 infection. Our ultimate goal was to elucidate how the C-terminal region of POM121C (i.e., POM987) interacts with viral proteins to inhibit HIV-1 infection. Interestingly, passaging HIV-1 in the presence of increasing amounts of POM987-expressing cells resulted in the outgrowth of viral escape mutants. We identified two amino acids in the HIV-1 CA protein that accounted for the resistance to POM987. These escape mutations disrupted the interaction between the assembled capsid and POM987, suggesting that the POM987-mediated inhibition of viral infectivity involves the physical interaction with the HIV-1 capsid core. The residues in CA necessary for this interaction were not previously known to be critical for nuclear pore interaction. Our study therefore provides novel mechanistic insights into the nuclear import of HIV-1 preintegration complexes.

## RESULTS

### N-terminally truncated POM121C inhibits HIV-1 replication

**Fig 1:**
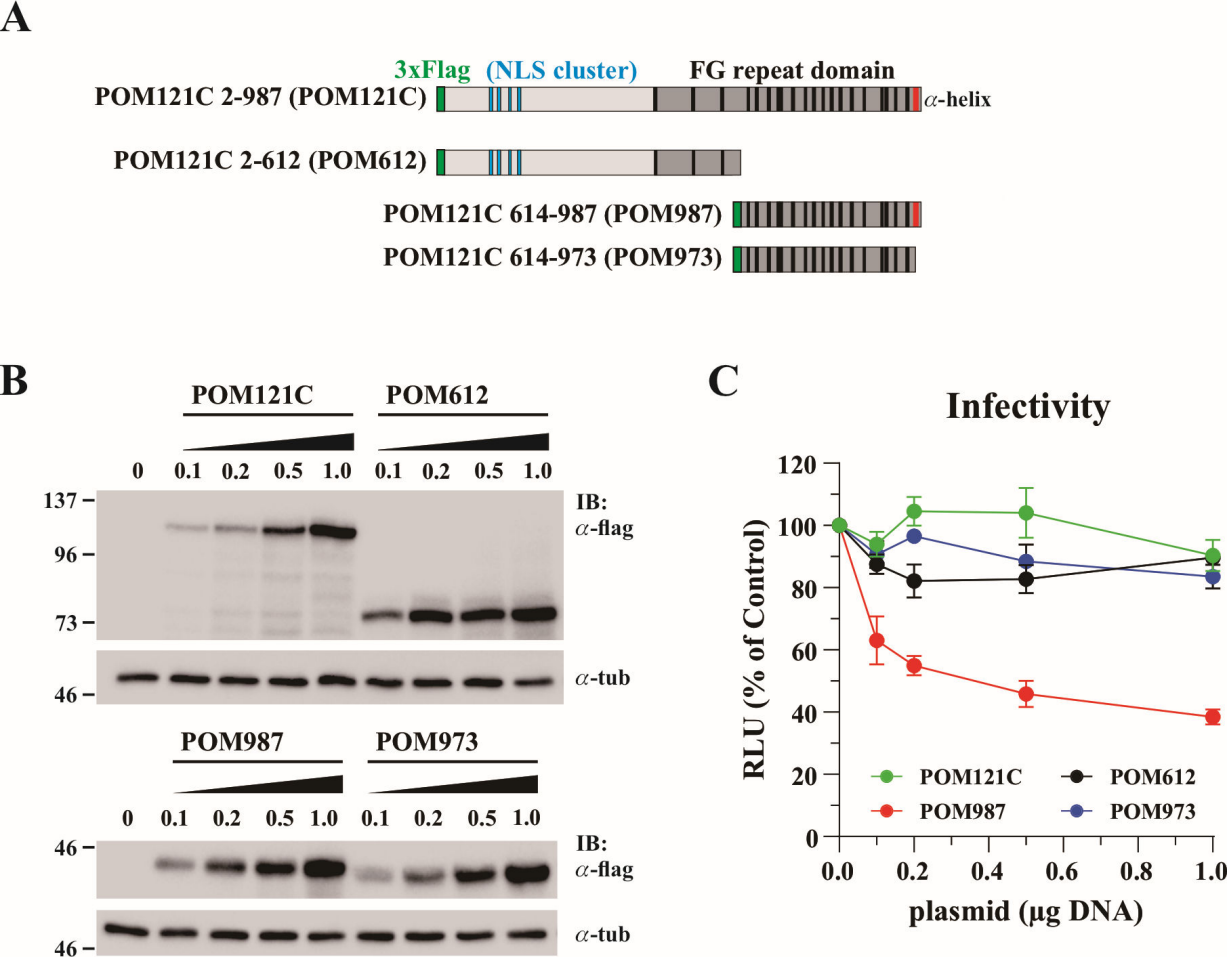
N-terminally truncated POM121C inhibits HIV-1 replication. (**A**) Schematic representation of POM121C (Gene ID:100101267) and its truncation mutants. Blue boxes indicate the nuclear localization signal domain (NLS), black boxes indicate the FG-repeat domain, red boxes mark a predicted α-helix motif, and green boxes represent the N-terminal 3xFlag epitope tag. (**B, C**) HEK293T cells were transfected with increasing amounts of p3xFlag-POM121C, p3xFlag-POM612, p3xFlag-POM987, or p3xFlag-POM973 (0, 0.1, 0.2, 0.5, and 1.0 µg, respectively). Total transfected plasmid DNA was adjusted to 2.0 µg per sample using empty vector DNA. (**B**) Cells were harvested 24 h post-infection and analyzed by immunoblotting for protein expression using antibodies to the Flag epitope. Expression of tubulin served as a control for equal sample loading. (**C**) HEK293T cells were transfected as described in panel B. The next day, cells were reseeded into a 24-well plate and infected with equal amounts of VSV-G-pseudotyped NL4-3-luc. Luciferase activity was measured 48 h post-infection as relative luminescence units (RLU), and values for the empty-vector control were set to 100%. Results for cells expressing POM121C (2-987), POM121C (2-612)/POM612, POM121C (614-973)/POM973, and POM121C (614-987)/POM987 are shown in the indicated colors. Data represent the mean ± SEM from three independent experiments.

We previously reported that the C-terminal region of POM121C inhibits HIV-1 infection (46). More recently, it was reported that POM121A is an essential factor for HIV-1 infection (47). It was also shown that expression of a truncated C-terminal region of POM121A inhibits HIV-1 infection (48). However, as with POM121C, the underlying mechanism of interference remained unclear. To establish a functional link between expression of truncated POM121C and the antiviral effect, we constructed several additional vectors expressing various truncated forms of POM121C. All POM121C constructs were designed to carry an N-terminal Flag-tag. POM987, which encodes residues 614–987, was previously identified as an HIV-1 inhibitory fragment, whereas POM612, which encodes residues 2–612, was included to examine the contribution of the N-terminal region of POM121C. POM973, which encodes residues 614–973 and lacks only the C-terminal 14 amino acids of POM987, was previously shown to lack anti-HIV-1 activity (46) and therefore served as an informative negative control (Fig. 1A).

We first tested transient expression of these constructs by transfecting increasing amounts of POM vectors into HEK293T cells followed by immunoblotting using an antibody to the N-terminal Flag epitope. We found that all four constructs efficiently expressed the encoded protein in a dose-dependent manner (Fig. 1B), confirming that deletions within POM121C had no effect on protein expression or stability. To verify reported effects of POM overexpression on cellular susceptibility to HIV-1 infection, HEK293T cells were transfected with increasing amounts of POM vectors as in panel B. One day later, transfected cells were detached and reseeded into three wells of a 12-well plate. Cells were then exposed for 24 h to VSV-G-pseudotyped NL4-3-luc reporter virus (Fig. 1C). Consistent with our previous results (46), we found that exogenous expression of POM121C, POM612, and POM973 had no effect on the sensitivity of HEK293T cells to infection by NL4-3-luc virus (Fig. 1C). Only POM987 inhibited the infection of HEK293T cells by NL4-3-luc virus in a dose-dependent manner (Fig. 1C; red graph). It is important to note that the POM987 and POM973 constructs are identical except for the lack of 14 residues from the C-terminus in POM973 (Fig. 1A). These results are therefore consistent with our previous findings (46) and reinforce the notion that expression of the N-terminal 612 residues of POM121C (POM612) as well as expression of a C-terminal fragment encompassing residues 614–973 (POM973) does not compromise nuclear pore function and does not abolish the ability of HIV-1 to enter the nucleus and initiate viral gene expression. Our results also confirm that expression of POM987 rendered HEK293T cells resistant to infection by HIV-1. The experiments described here involved VSV-G pseudotyped virus that rules out an effect of POM987 on virus entry and therefore more likely reflects a post-entry function of POM987 on the early steps of HIV-1 infection in the target cell.

### POM987 and POM973 predominantly localize to the cytoplasm but differ in antiviral activity

**Fig 2:**
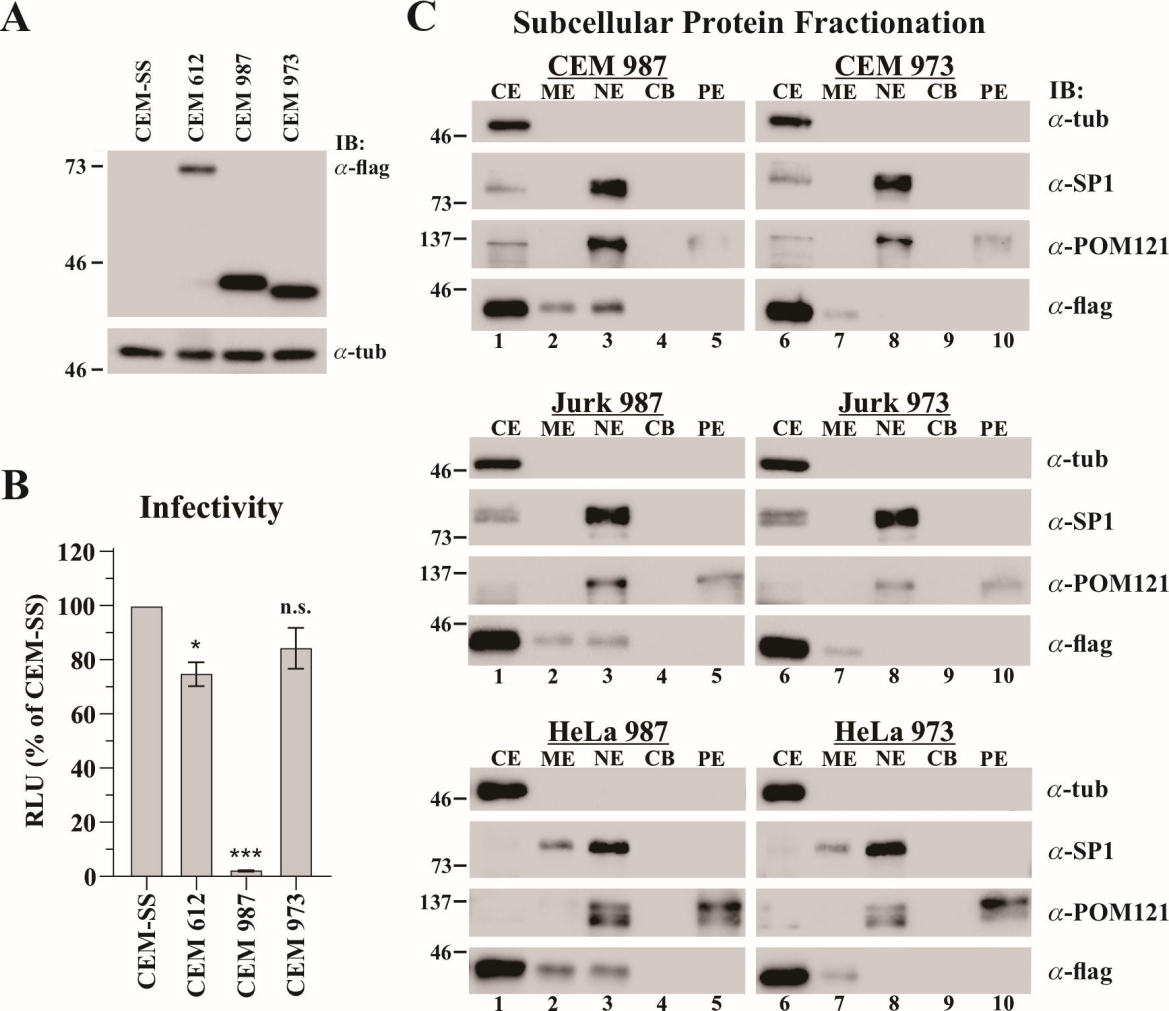
Overexpression of POM987 but not POM973 induces resistance to HIV-1 infection. (**A**) CEM-SS cells were stably transduced with VSV-G-pseudotyped lentiviral vectors expressing POM121C (2-612)/POM612, POM121C (614-987)/POM987, and POM121C (614-973)/POM973 and selected with 2 µg/mL puromycin. The resulting cell lines are referred to as CEM 612, CEM 987, and CEM 973, respectively. Expression of the Flag-tagged POM variants was assessed by immunoblotting as described in Fig. 1B. (**B**) CEM-SS, CEM 612, CEM 987, and CEM 973 cells were infected with VSV-G-pseudotyped NL4-3-luc and harvested 48 h post-infection. Luciferase activity produced in parental CEM-SS cells was set to 100%. Data represent the mean ± SEM from six independent experiments. The statistical significance was determined using one-way ANOVA analysis with a Dunnett’s multiple comparisons test. *P < 0.05, ***P < 0.001, n.s: not significant. (**C**) Subcellular localization of stably expressed POM987 and POM973 in CEM-SS, Jurkat, and HeLa cells was analyzed using a commercial subcellular fractionation kit (Thermo Fisher Scientific, Waltham, MA, Cat# 78840). The corresponding stable cell lines are indicated as CEM 987, CEM 973, Jurk 987, Jurk 973, HeLa 987, and HeLa 973. Cells were fractionated into cytoplasmic extract (CE), membrane extract (ME), soluble nuclear extract (NE), chromatin-bound (CB), and pellet extract (PE) fractions. Immunoblotting was performed using antibodies against tubulin (cytoplasmic marker), SP1 (nuclear marker), POM121 (endogenous wild-type POM121A), and the Flag tag in POM987 or POM973. A representative result from three independent experiments is shown.

To better understand the significance of the 14 C-terminal residues of POM121C during HIV-1 infection, we created CEM-SS cell lines stably expressing N-terminally Flag-tagged POM612, POM987, and POM973. In the following, we refer to these cell lines as CEM 612, CEM 987, and CEM 973, respectively. Protein expression was verified by immunoblotting (Fig. 2A). Attempts to create a stable cell line expressing Flag-tagged full-length POM121C 2-987 were unsuccessful. Therefore, pursuing the analysis of POM 2-987 was technically challenging. Resistance of stable POM cell lines to HIV-1 infection was determined by infection with VSV-G-pseudotyped NL4-3-luc virus. Consistent with the results from Fig. 1C, CEM 987 cells but not CEM 612 or CEM 973 cells exhibited resistance to HIV-1 infection (Fig. 2B). The stronger inhibition observed in stable CEM 987 cells compared with transiently transfected 293T cells likely reflects differences in expression systems, as stable expression of POM987 in 293T cells also resulted in robust inhibition of HIV-1 infection (46). Because of the close similarity of POM987 and POM973 and the lack of effect of POM612 in the stable CEM-SS cells, the remainder of this study focused on a comparison of POM987 and POM973.

To identify possible differences in the intracellular distribution of POM987 and POM973, we performed a cell fractionation analysis of CEM 987 and CEM 973 cells using a commercial subcellular protein fractionation kit. Cells were fractionated into CE, ME, soluble NE, CB, and PE fractions and analyzed by immunoblotting. Cytoplasmic (tubulin) and nuclear (SP1) markers served as controls. Endogenous POM121 was identified using a POM121 polyclonal peptide antibody, whereas the POM987 and POM973 translation products were identified using a Flag antibody (Fig. 2C). Consistent with its predicted localization to the nuclear pores, endogenous POM121 was restricted largely to the soluble NE fraction. Indeed, the cellular distribution of the endogenous POM121 closely matched that of the nuclear transcription factor SP1. Not unexpectedly, POM987 and POM973 dramatically differed from endogenous POM121 in their subcellular localization. Both truncated proteins largely partitioned into the cytoplasmic fraction (Fig. 2C; lanes 1 and 6), consistent with the lack of an NLS. POM973 localized almost exclusively to the cytoplasmic fraction, whereas a small portion of POM987 was detected in the nuclear fraction (Fig. 2C; lane 3). This pattern was reproducibly observed in CEM-SS, Jurkat, and HeLa cells (Fig. 2C). Although the predicted C-terminal α-helix motif present in POM987 but absent from POM973 (Fig. 1A) may contribute to this subtle difference in fractionation, both proteins predominantly localized to the cytoplasmic fraction.

### POM121A isoform 2 but not isoform 1 induces viral resistance in CEM-SS cells

**Fig 3:**
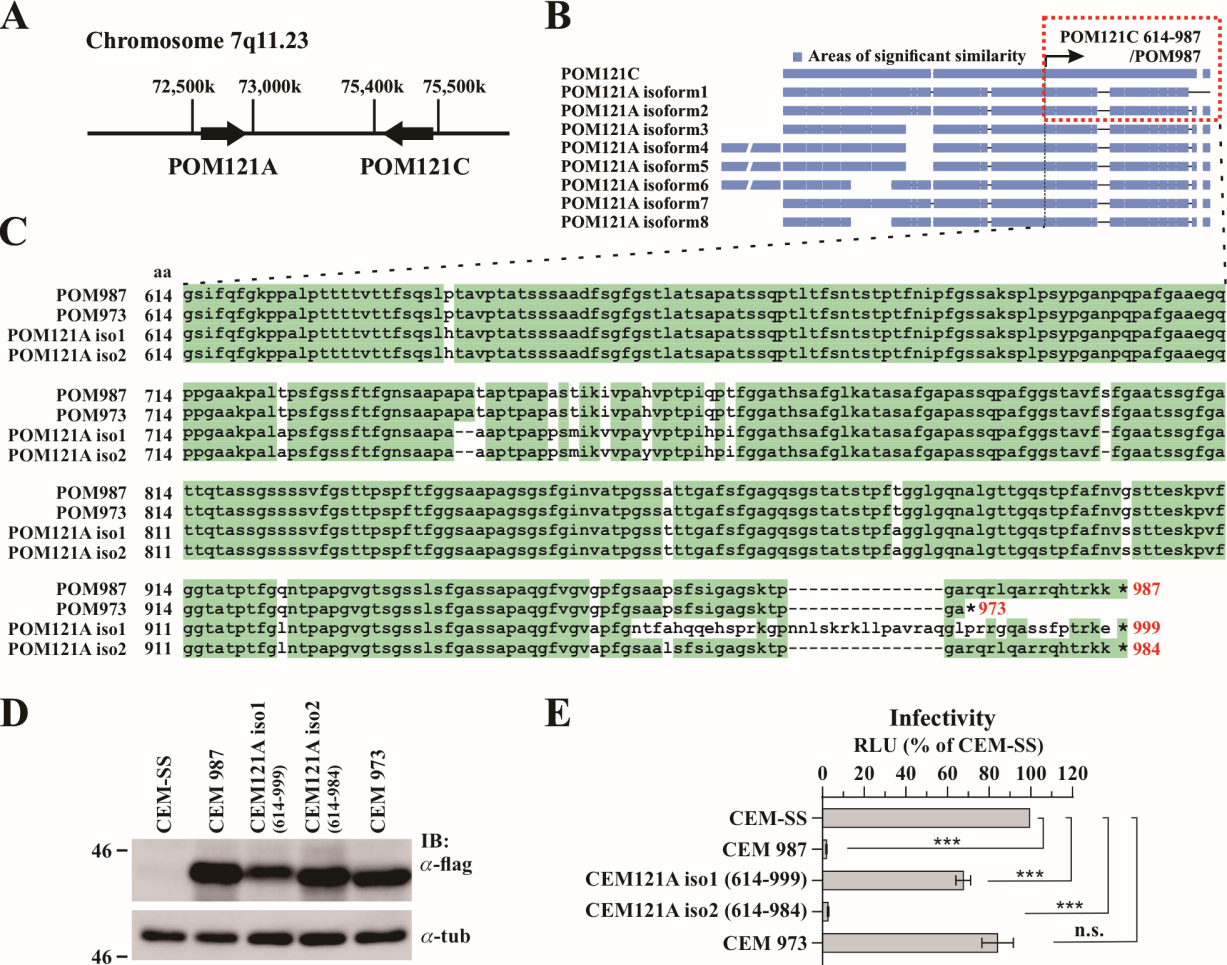
A C-terminal motif in POM121C/POM121A isoform2 is critical for inhibition of HIV-1 infection. (**A**) POM121 (referred to in the following as POM121A) (Gene ID: 9883) and POM121C (Gene ID:100101267) genes are located on human chromosome 7. (**B**) POM121C exists in only one isoform, while POM121A has eight isoforms. The amino acid alignment is shown schematically. The blue regions indicate sequence similarity. White areas indicate in-frame amino acid deletions. The area enclosed by the red dotted line represents the expression region of the truncated protein in this study. (**C**) Amino acid alignment of POM121C (614–987)/POM987, POM121C (614–973)/POM973, POM121A isoforms 1 (614–999), and POM121A isoforms 2 (614–984). Green areas mark regions of amino acid identity. Dashes indicate amino acid deletions. (**D and E**) The C-terminal region of POM121C is critical for inducing resistance to HIV-1 in CEM-SS cells. CEM-SS cells were transduced with a VSV-G-pseudotyped lentiviral vector expressing POM987, POM973, the C-terminal regions of POM121A isoform 1 (614–999), and POM121A isoform 2 (614–984). The resulting cell lines are referred to as CEM 987, CEM 973, CEM121A iso1 (614–999), and CEM121A iso2 (614–984), respectively. (**D**) Cell extracts from stable puromycin-selected cultures were subjected to immunoblot analysis using antibodies against the Flag tag and tubulin to verify comparable expression of POM variants. (**E**) Stably transduced cells were then infected with VSV-G pseudotyped NL4-3-luc reporter virus as in Fig. 2B and luciferase activity was measured 48 h post-infection. Luciferase activity in parental CEM-SS cells was set to 100%. Data represent the mean ± SEM from three independent experiments. The statistical significance was determined using one-way ANOVA analysis with a Dunnett’s multiple comparisons test. ***P < 0.001, n.s.: not significant.

Unlike rodents, which carry a single POM121 gene, human cells possess multiple POM121 gene loci on chromosome 7 (7q11.23; Fig. 3A) (43). Notably, POM121C has only one known translation product, whereas POM121A has eight isoforms (Fig. 3B). POM121A isoforms 2–8 are splice variants whose amino acid sequences downstream of residue 614 are identical. For comparison, we aligned the amino acid sequences of POM121C (starting at residue 614) to those of the POM121A isoforms 1 and 2 (as representative for isoforms 2–8). As shown in Fig. 3C, the sequences of POM121C (POM987) and POM121A isoform 2 are very similar. POM121A isoform 1, on the other hand, differs from isoform 2 and POM121C at the C-terminus. Of note, POM121A isoform 1 differs from isoforms 2–8 and POM121C by the presence of a 15 amino acid insertion and multiple additional amino acid changes near the C-terminus. Interestingly, POM121C and POM121A isoform 2 are identical at the 27 C-terminal residues (Fig. 3C). Based on our results with the POM121C variants in Fig. 1, we predicted that N-terminally truncated POM121A isoforms 2–8 but not isoform 1 would exert antiviral activities. To test this hypothesis, we created stable CEM-SS cell lines expressing POM121A isoforms 1 (614–999) and isoforms 2 (614–984), with isoform 2 acting as a surrogate for isoforms 3–8. For consistency, POM121A isoforms 1 and 2 were designed to encode residues 614 to their C-termini with an N-terminal Flag-tag. Stable CEM-SS cell lines for expression of isoforms 121 iso1 (614–999) and 121 iso2 (614–984) were referred to as CEM121 iso1 (614–999) and CEM121 iso2 (614–984). Protein expression relative to CEM 973 and CEM 987 cells was assessed by immunoblotting (Fig. 3D). Infection of CEM121 iso1 (614–999) and CEM121 iso2 (614–984) cells was done in parallel with infection of CEM 987 and CEM 973 cells (Fig. 3E). The results indeed showed that CEM121 iso2 (614–984) but not CEM121 iso1 (614–999) expressing cells restrict infection by HIV-1 NL4-3, thus supporting our hypothesis.

### Passaging of HIV-1 in CEM987 cells leads to outgrowth of viral escape mutants

**Fig 4:**
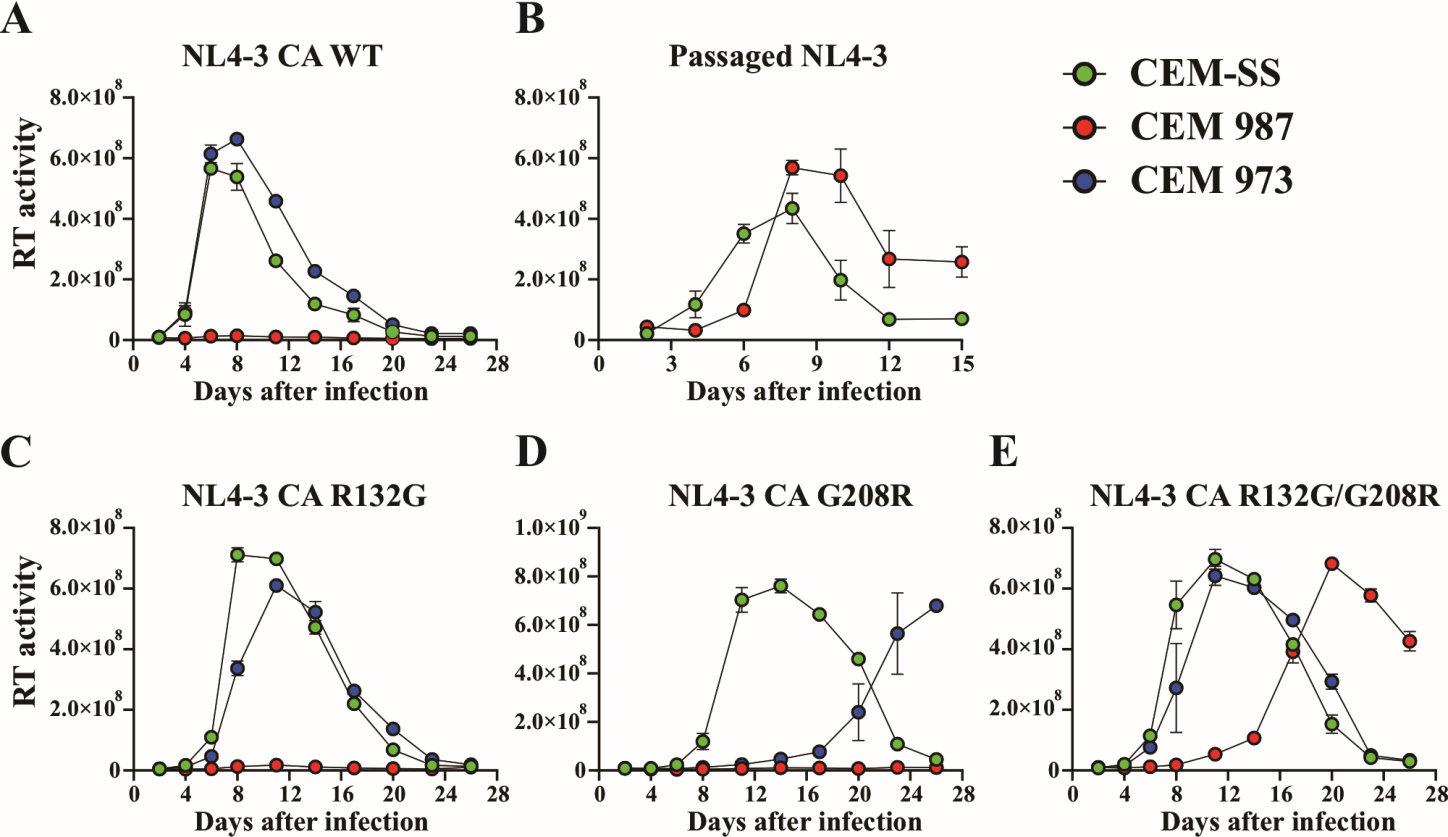
Identification of escape mutations that interfere with the antiviral activity of POM987. (**A**) Parental CEM-SS cells as well as CEM 987 and CEM 973 cells were infected with wild-type NL4-3. Virus replication was monitored by measuring the release of virus-associated reverse transcriptase (RT) over time. Mean and error bars representing SEM calculated from triplicate infections are shown. (**B**) Serial passaging of NL4-3 in a 10:1 starting mixture of parental CEM-SS and CEM 987 cells. The proportion of CEM 987 to parental CEM-SS cells was gradually increased with each passage. Shown is the replication profile after the 7th passage. Error bars represent the mean and SEM from triplicate infections. (**C–E**) Parental CEM-SS cells, as well as CEM 987 or CEM 973 cells, were infected with NL4-3 carrying specific CA mutations. Shown are the replication profiles of viruses carrying single amino acid changes in (**C**) CA R132G and (**D**) CA G208R. (**E**) Replication profile of the CA R132G/G208R double mutant. Mean and error bars representing SEM calculated from triplicate infections are shown.

To determine which viral functions are targeted by POM987, we set out to isolate viral escape mutants capable of replicating in CEM 987 cells. Initial attempts to establish a spreading infection of wild-type NL4-3 in CEM 987 cells failed due to the strong antiviral effect of POM987 (Fig. 4A; red graph). As expected, cells expressing POM973 did not restrict HIV-1 infection (Fig. 4A; compare green and blue graphs). Since HIV-1 NL4-3 replicates well in the parental CEM-SS cells (Fig. 4A; green graph), we mixed CEM-SS and CEM 987 cells initially at a 10:1 ratio and then infected the mixed cell population with NL4-3 wt. Virus was passaged multiple times in cultures containing gradually increasing proportions of CEM 987 cells, up to 100%. After seven virus passages, we obtained a virus population capable of replicating in straight CEM 987 cells (Fig. 4B; red graph). Sequence analysis of viral RNA isolated from the escape virus population revealed two amino acid changes in the CA region of the viral Gag protein, R132G and G208R. These mutations were identified by direct Sanger sequencing of bulk RT-PCR products and confirmed by sequencing individual PCR clones. No additional consensus-level mutations were detected in the other regions of the viral genome. We constructed NL4-3 molecular clones carrying the R132G (Fig. 4C) and G208R mutations (Fig. 4D) alone. In addition, we constructed a variant containing both amino acid changes (Fig. 4E). The resulting viruses were used to infect CEM 987 cells. We found that changing R132G or G208R alone was insufficient to restore virus replication in CEM 987 cells (Fig. 4C and D; red circles). Notably, the G208R single mutant replicated less efficiently in CEM 973 cells than in parental CEM-SS cells (Fig. 4D; blue and green circles), although POM973 did not restrict wild-type and R132G HIV-1 replication (Fig. 4A and C; blue circles). Both single- and double-mutant viruses replicated well in parental CEM-SS cells (Fig. 4C and D; compare red and green circles), indicating that the introduction of R132G or G208R mutations into the NL4-3 Gag protein itself did not impair viral fitness. Importantly, viruses containing both mutations in CA were able to establish a spreading infection in CEM 987 cells even though the kinetics of replication were still delayed when compared to parental CEM-SS cells (Fig. 4E; compare red and green graphs). We hypothesize that mutation of R132 and G208 induces structural changes in the viral capsid that render it resistant to interference by POM987.

### Viral escape from POM987-induced restriction is cell type independent

**Fig 5:**
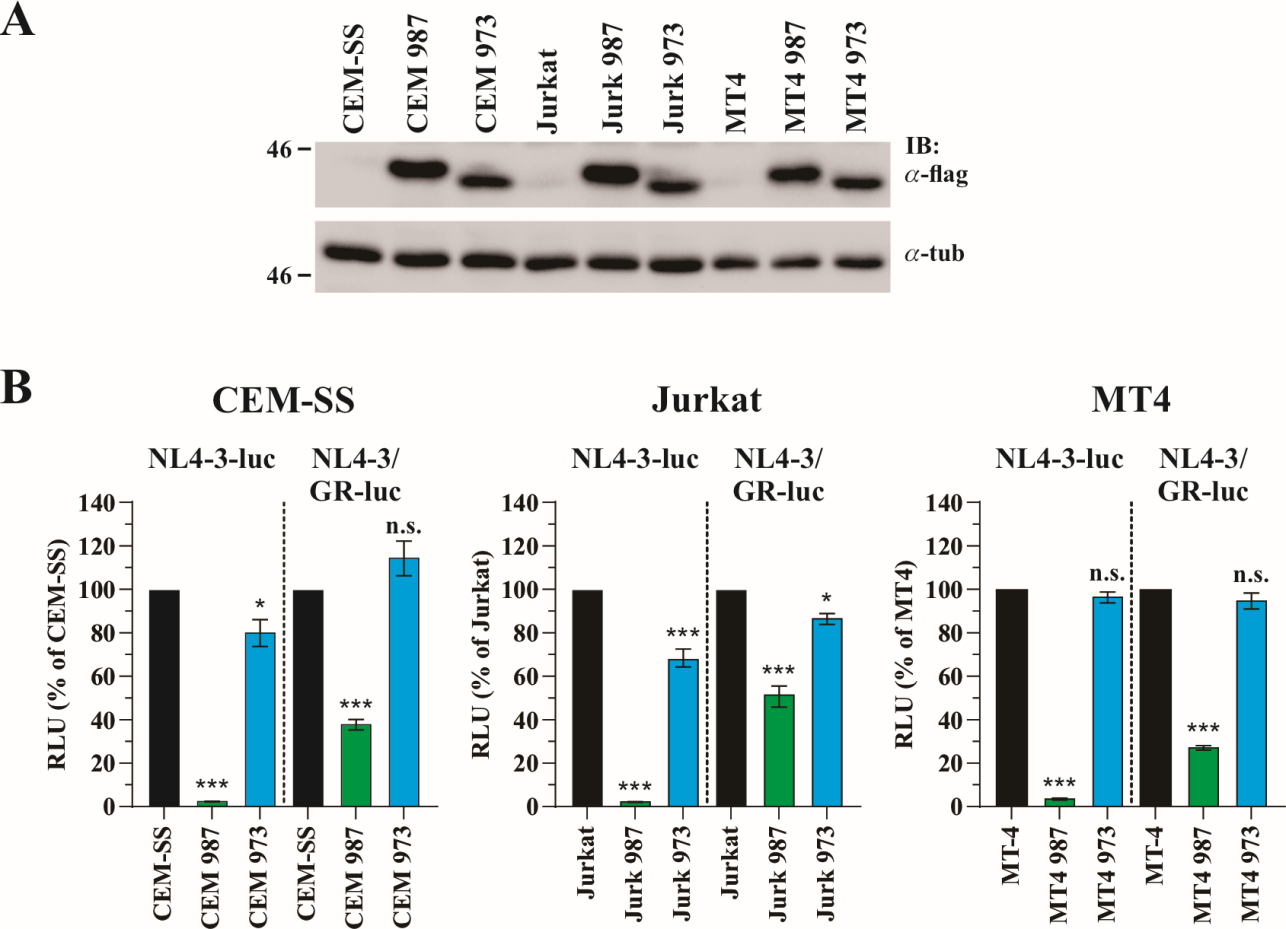
Viral escape is cell type independent. CEM-SS, Jurkat, and MT4 cells were transduced with a VSV-G-pseudotyped lentiviral vector expressing Flag-tagged POM987 or POM973. (**A**) Cell lysates were subjected to immunoblot analysis using antibodies against the Flag tag of POM121C truncated variants (top) and tubulin (bottom). (**B**) Cell lines established above were infected with VSV-G-pseudotyped NL4-3-luc reporter virus carrying CA WT (NL4-3-luc) or the CA double mutant R132G/G208R (NL4-3/GR-luc). Cells were harvested 48 h post-infection, and luciferase production was determined. Luciferase activity in parental cells was set to 100% each. Data represent the mean ± SEM from at least three independent experiments. The statistical significance was determined using one-way ANOVA analysis with a Dunnett’s multiple comparisons test. *P < 0.05, ***P < 0.001, n.s.: not significant.

To further characterize the properties of our CA escape mutant, we introduced the R132G and G208R mutations into the NL4-3-luc reporter virus. The resulting NL4-3/GR-luc virus was then used to test whether the escape phenotype was specific for CEM-SS cells or could be recapitulated in other cell types. Thus, we created Jurkat and MT4 cell lines stably expressing POM987 or POM973. Expression of the POM variants in these cells was verified by immunoblotting (Fig. 5A). Cells were infected with VSV-G pseudotyped NL4-3/GR-luc virus stocks, and luciferase production was measured 24 h later (Fig. 5B). Indeed, NL4-3-luc encoding wt capsid was severely restricted in CEM 987 cells as well as in Jurkat 987 (Jurk 987) and MT4 987 cells (Fig. 5B; green bars). As expected, expression of POM973 neither inhibited infection by NL4-3-luc nor by NL4-3/GR-luc (Fig. 5B; blue bars). In contrast, infection by NL4-3/GR-luc carrying the two capsid mutations revealed partial resistance to POM987 (Fig. 5B; green bars). A reason why there is no full reversal of the POM987 restriction could be the strong expression of POM987 in the transduced T-cell lines or could simply reflect incomplete resistance to POM987 in the NL4-3/GR escape mutant.

### POM987 expression interferes with the production of nuclear 2-LTR circles

**Fig 6:**
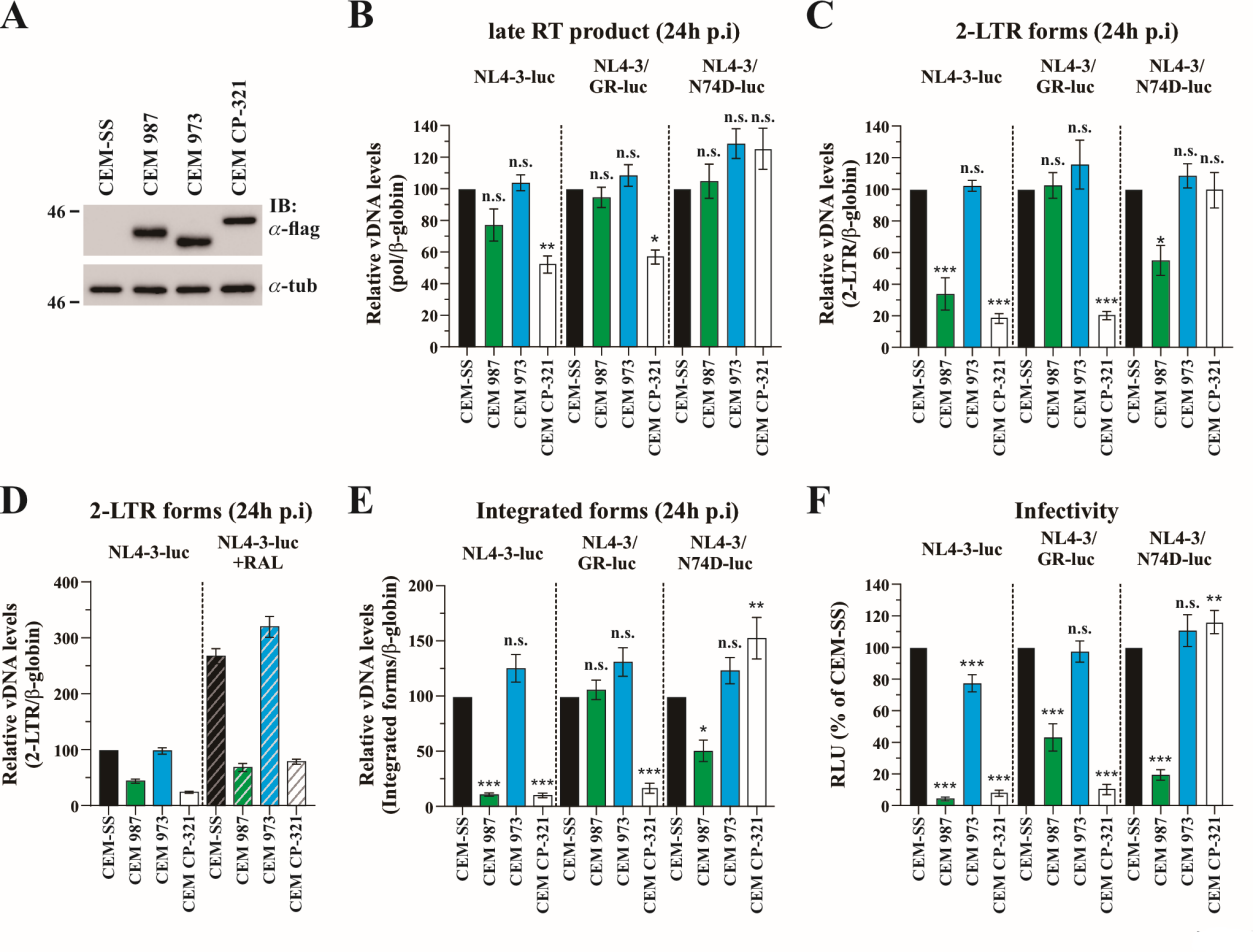
POM987 does not affect HIV-1 reverse transcription but inhibits 2-LTR circle formation. Cell lines CEM-SS, CEM 987, CEM 973, or Flag-tagged truncated CPSF6 (2–321) (CEM CP-321) were infected with VSV-G-pseudotyped NL4-3-luc reporter virus carrying CA WT (NL4-3-luc), CA double mutant R132G/G208R (NL4-3/GR-luc), or CA N74D mutant (NL4-3/N74D-luc). (**A**) Cell lysates were subjected to immunoblot analysis using antibodies against the Flag tag and tubulin. (**B, C, and D**) Relative amounts of viral cDNA synthesized after NL4-3luc infection. Total DNA was extracted 24 h post-infection and used for qPCR with primer sets recognizing the late reverse transcription (pol region) and 2-LTR circle. The integrated viral DNA was measured using an Alu-PCR method. The amount of DNA in each PCR assay was normalized for β-globin, and parental CEM-SS cells were set to 100%. Data represent the mean ± SEM from at least three independent experiments. (**E**) Viral infectivity in each cell type was measured as in Fig. 2B; luciferase activity observed in CEM-SS cells was defined as 100%. (**F**) CEM-SS, CEM 987, CEM 973, and CEM CP-321 cells were infected with VSV-G-pseudotyped NL4-3-luc in the absence or presence of 100 nM raltegravir (RAL), and 2-LTR circles were quantified by qPCR. Filled bars indicate infection in the absence of RAL, whereas striped bars indicate infection in the presence of RAL. Data represent the mean ± SEM from three independent experiments. The statistical significance was determined using one-way ANOVA analysis with a Dunnett’s multiple comparisons test. *P < 0.05, **P < 0.01, ***P < 0.001, n.s.: not significant.

Our finding that mutations in CA can induce resistance to POM987 could indicate that POM987 interacts with the viral capsid and/or competes with endogenous POM121C (anchoring the nuclear pore complexes in the nuclear membrane) for a critical function required for nuclear import. The predominantly cytoplasmic localization of POM987, together with its partial association with the nuclear fraction (Fig. 2C), could suggest that POM987 acts on the incoming virus particle and somehow interferes with nuclear import. Here, we assessed the effects of POM987 and POM973 on the synthesis of late RT products as well as 2-LTR circles as a marker associated with nuclear entry. As a control, we included CPSF6-321, which interferes with the nuclear import of HIV-1 but can be neutralized by mutation of N74D in the viral capsid (14). Thus, we created a CEM-SS cell line stably expressing Flag-tagged CPSF6-321 (CEM CP-321). Protein expression was verified by immunoblotting (Fig. 6A). Stably transduced CEM CP-321 cells, together with parental CEM-SS cells, or POM-expressing CEM 987 or CEM 973 cells, were infected with NL4-3-luc, NL4-3/GR-luc, or NL4-3/N74D-luc. Cells were collected 24 h after infection and processed for qPCR to detect and quantify viral DNA products (Fig. 6B and C). In parallel, viral infectivity was measured by luciferase assay (Fig. 6F). As expected, expression of POM973 did not affect the synthesis of late RT products, nor did it inhibit the formation of 2-LTR circles (Fig. 6B and C; blue bars). POM973 also did not inhibit the infectivity of the POM987 escape mutant (NL4-3/GR-luc) and did not inhibit the infectivity of the CPSF6-321 escape mutant (NL4-3/N74D-luc) (Fig. 6F; blue bars). POM973 did cause a slight albeit insignificant reduction in the infectivity of NL4-3-luc. As expected, expression of CPSF6-321 reduced late reverse transcription products, 2-LTR circle formation, and infectivity of NL4-3-luc and NL4-3/GR-luc, but not the infectivity of the CPSF6-321 escape mutant NL4-3/N74D-luc (Fig. 6B, C, and F; white bars). Finally, expression of POM987 did not, or only modestly, interfere with reverse transcription of incoming viruses (Fig. 6B; green bars). Importantly, POM987 strongly inhibited the formation of 2-LTR circles by NL4-3-luc, but not by the escape mutant NL4-3/GR-luc (Fig. 6C; green bars). Consistent with this reduction in nuclear viral DNA, Alu-PCR analysis showed that integrated viral DNA was markedly reduced in CEM 987 cells infected with NL4-3-luc, whereas integration of the escape mutant NL4-3/GR-luc was restored to levels comparable to those observed in parental CEM-SS cells (Fig. 6E). To further distinguish whether the reduction in integrated viral DNA reflected impaired nuclear entry or a direct block to integration, we quantified 2-LTR circles in the absence or presence of the integrase inhibitor raltegravir (RAL). In parental CEM-SS cells and CEM 973 cells, RAL treatment resulted in the expected accumulation of 2-LTR circles. In contrast, 2-LTR circle levels remained low in CEM 987 cells even in the presence of RAL (Fig. 6D). Although NL4-3/GR-luc restored 2-LTR circle formation and integrated viral DNA levels in CEM 987 cells, luciferase activity was only partially restored (Fig. 6F). The basis for this discrepancy remains unclear, but it may reflect effects on steps downstream of integration or differences between integrated viral DNA levels and reporter gene expression. Together, these results suggest that POM987 primarily impairs HIV-1 nuclear entry, which likely accounts for the subsequent reduction in integrated viral DNA and infectivity.

### POM987 inhibits nuclear entry of viral capsids

**Fig 7:**
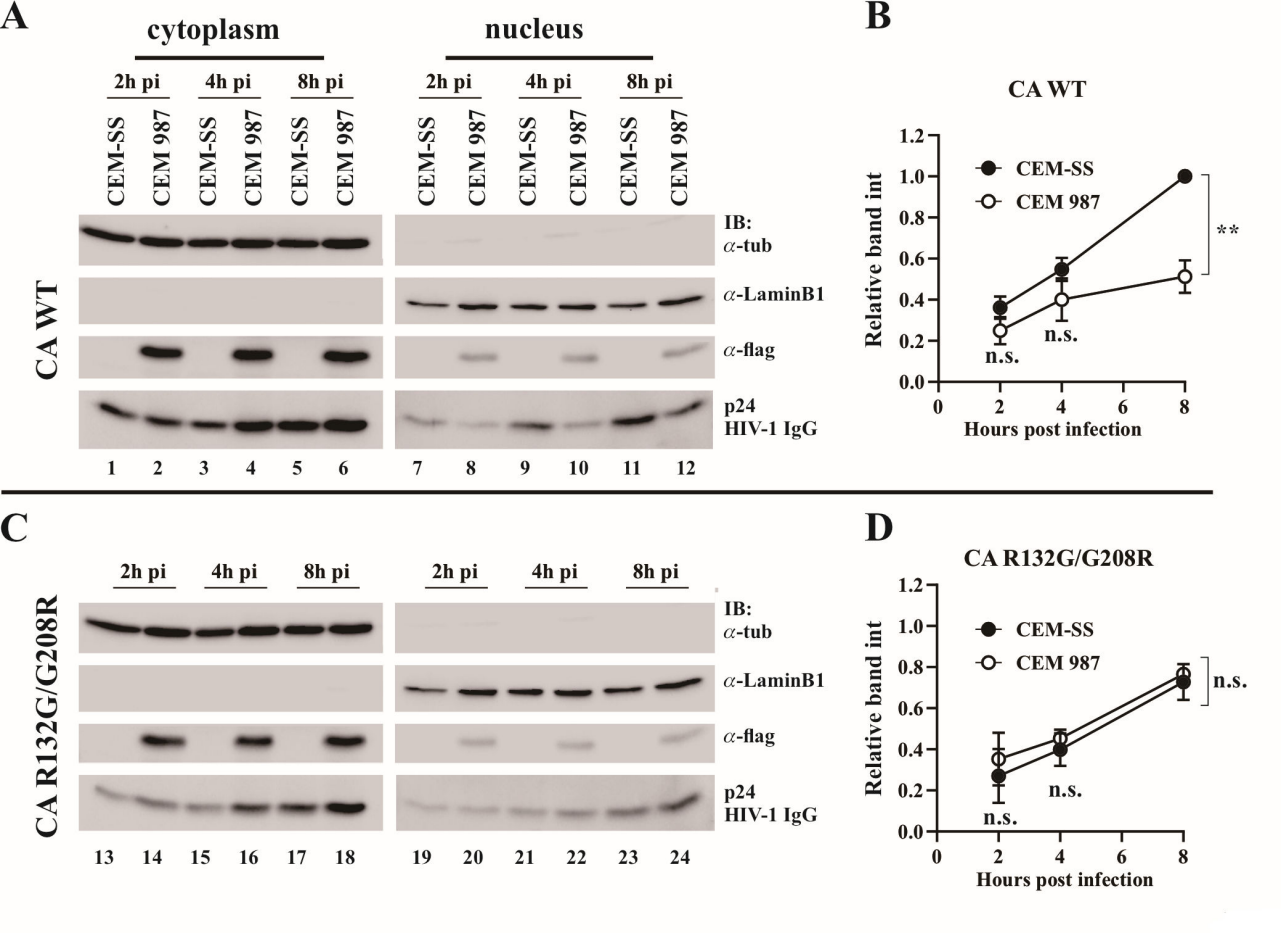
POM987 inhibits nuclear entry of HIV-1 capsids. (**A and C**) CEM-SS and CEM 987 cells were infected with VSV-G-pseudotyped NL4-3-luc CA WT or NL4-3-luc CA R132G/G208R. To inhibit newly synthesized viral proteins, an integrase inhibitor (100 nM RAL) was added to the culture medium. At the indicated time points, cell extracts were separated into cytoplasmic and nuclear fractions using a commercial fractionation kit (Thermo Fisher Scientific, Waltham, MA, Cat# 78835). p24 capsid content in each fraction was analyzed by immunoblotting (p24; HIV-1 IgG). Expression of POM987 was verified by probing with antibodies to the Flag epitope. Tubulin was included as a marker for cytoplasmic proteins, and LaminB1 served as a nuclear marker. (**B and D**) Capsid (P24) band intensity in nuclear fractions was quantified using Image Lab software (Bio-Rad). Capsid band intensity observed in NL4-3-luc-infected CEM-SS at the 8-h time point was defined as 1. Data represent the mean ± SEM from three independent experiments. The statistical significance was determined using one-way ANOVA analysis with a Dunnett’s multiple comparisons test. **P < 0.01, n.s.: not significant.

To further investigate the possibility that POM987 affects nuclear import of wild-type NL4-3-luc, we focused on the viral nuclear import process and conducted a capsid nuclear import assay as reported (31). CEM-SS cells or CEM 987 cells were infected with NL4-3-luc (Fig. 7A and B) or NL4-3/GR-luc (Fig. 7C and D). Cells were collected at 2, 4, or 8 h after infection and separated into cytoplasmic and nuclear fractions to track the bulk nuclear import of viral capsid using a biochemical assay. In CEM-SS cells infected with NL4-3-luc, nuclear capsid protein levels in the nuclear fraction increased over time (Fig. 7A; lanes 7, 9, and 11). In CEM 987 cells, nuclear capsid levels also increased with time (Fig. 7A; lanes 8, 10, and 12) but at a much reduced rate (Fig. 7B; compare open and closed circles). On the other hand, infection of CEM-SS and CEM 987 cells with NL4-3-luc and NL4-3/GR-luc did not reveal any differences between the viruses (Fig. 7C; compare lanes 19, 21, and 23 to lanes 20, 22, and 24 and Fig. 7D; compare open and closed circles). Of note, in the fractionation kit used in this study, proteins in the nuclear membrane were included in the nuclear fraction, which was confirmed by LaminB1 (Fig. 7A and C; lanes 7–12 and 19–24). Tubulin served as a cytoplasmic marker (Fig. 7A and C, lanes 1–6 and 13–18). POM987 accumulated in the cytoplasmic fraction but could also be found in the nuclear fractions (Fig. 7A and C; a-flag). Since capsid in the nuclear fraction of CEM 987 cells was reduced when compared to CEM-SS cells (Fig. 7A, compare lanes 11 and 12 and Fig. 7B, compare open and closed circles), our results suggest that POM987 may sequester the incoming HIV-1 core in the cytoplasm.

### The binding of POM987 to the HIV-1 core is important for its ability to block HIV-1 infection

**Fig 8:**
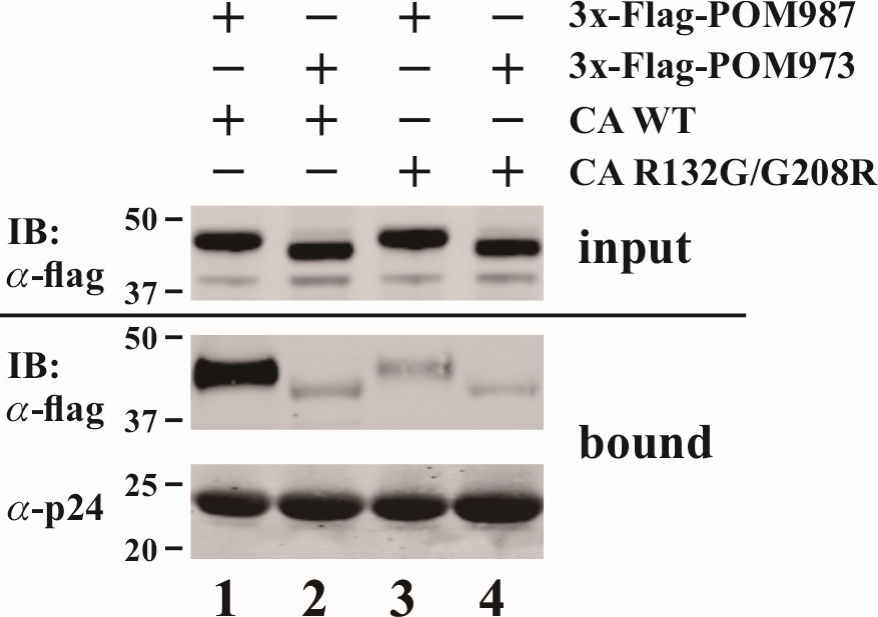
The C-terminal region of POM121C is required for binding to the HIV-1 core. To determine the binding of Flag-tagged POM987 or POM973 to stabilized HIV-1 capsid (A14C/E45C) tubes as previously described (49), HEK293T cells were transfected with plasmids expressing Flag-tagged POM987 or POM973. The cells were lysed (input) and incubated for 1 h with CA WT or CA R132G/G208R stabilized HIV-1 capsid tubes. Subsequently, tubes were washed with PBS, stabilized HIV-1 capsid tubes were pelleted at 21,000 × *g*, and resuspended in 1x Laemmli buffer (bound). Input and bound fractions were analyzed by immunoblotting using anti-Flag and anti-p24 antibodies. Detailed experimental procedures are described in Materials and Methods. A representative result from three independent experiments is shown.

Given that the C-terminal domain of POM121A interacts with the HIV-1 core (48), and that HIV-1 viruses bearing mutations in the capsid overcome the block imposed on HIV-1 by POM987, it seems reasonable to propose that the ability of POM987 to block HIV-1 infection is the result of an interaction between POM987 and the HIV-1 core. To this end, we tested the ability of POM987 and POM973 derived from cell lysates to bind to stabilized HIV-1 capsid tubes as previously described (49). In agreement with a model in which POM987 blocks HIV-1 infection by interaction with the HIV-1 core, we found that POM987 binds to wild-type stabilized HIV-1 capsid tubes (Fig. 8; lane 1). By contrast, POM973, which is unable to block HIV-1 infection, did not bind to HIV-1 stabilized capsid tubes (Fig. 8; lane 2). These experiments correlate inhibition of HIV-1 infection by POM987 with its ability to bind to the HIV-1 core. Furthermore, POM987 fails to bind to stabilized HIV-1 capsid tubes bearing the R132G/G208R mutations (Fig. 8; lane 3), which render HIV-1 infection resistant to POM987. These results further support the notion that inhibition of infection by POM987 occurs through binding to the HIV-1 core. Overall, these results suggest that POM987 binds to the HIV-1 core and inhibits nuclear import of the HIV-1 core during infection.

### Location of the escape mutations in HIV-1 capsid core

**Fig 9:**
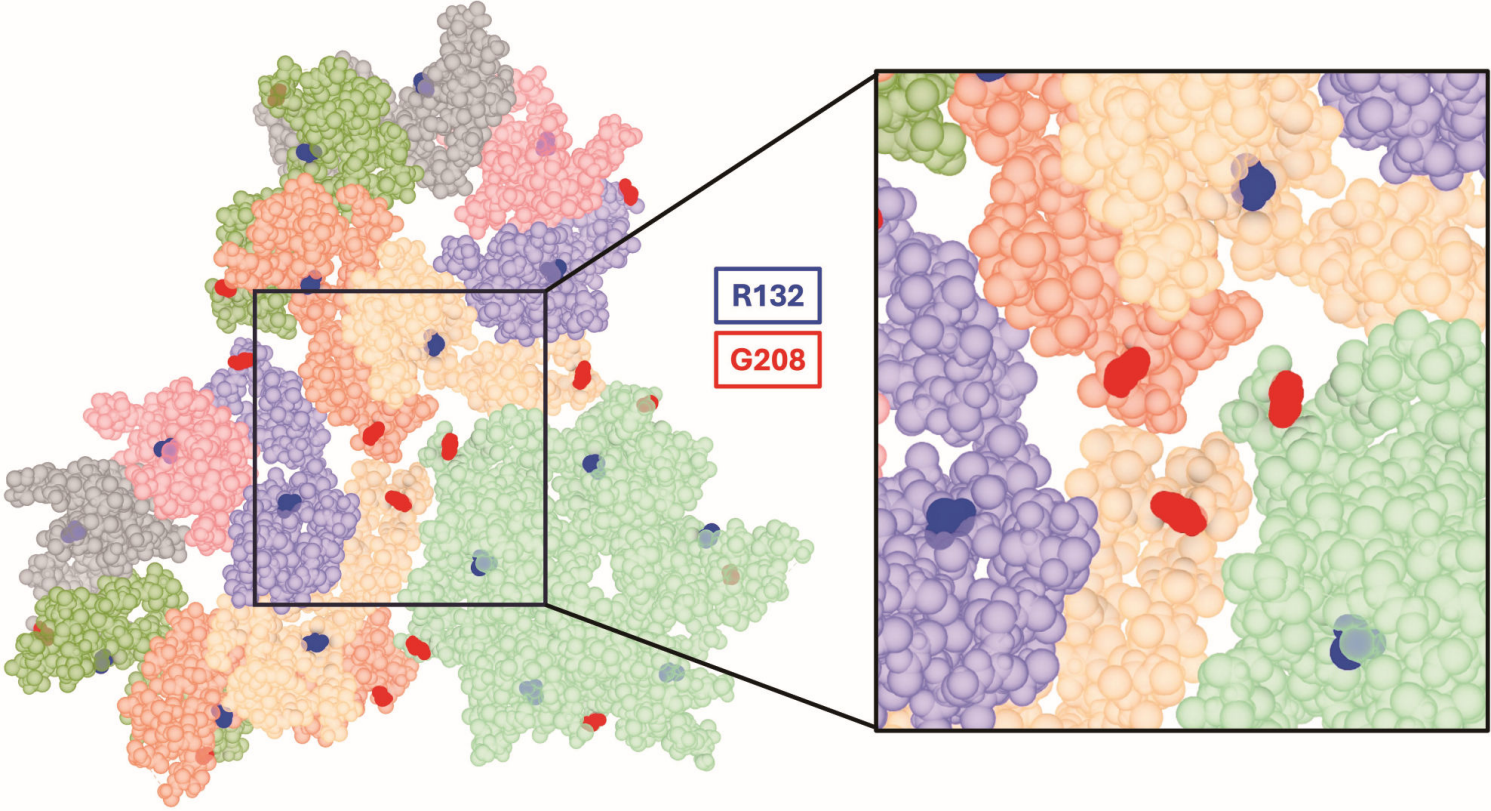
Visualizing POM987 escape mutations along the HIV-1 capsid lattice. The hexameric and pentameric CA subunits are shown on the left (PDB 8G6K) in space-filling atom representation. The two hexamers are colored by chain, and the pentamer is shown in light green. A magnified view of the trimer interface involving two CA hexamers and one CA pentamer is shown on the right. R132 is highlighted in blue, and G208 is highlighted in red. Molecular graphics and color changes were performed with ChimeraX.

To visualize the positions of the POM987 escape mutations within the assembled HIV-1 capsid core, R132 and G208 were mapped onto the HIV-1 capsid structure (Fig. 9). In this model, G208 is located at a trimer interface formed by two CA hexamers and one CA pentamer, whereas R132 is positioned in the CA N-terminal domain away from this interface. These two residues therefore occupy structurally distinct regions of the capsid core. However, the structural and functional consequences of the R132G and G208R substitutions on the assembled capsid core remain unclear. Further structural and biochemical analyses will be required to determine how these mutations affect capsid core properties and contribute to escape from POM987-mediated restriction.

## DISCUSSION

In this study, we investigated how POM987, a C-terminal fragment of POM121C, inhibits HIV-1 infection. Our results show that POM987 inhibits HIV-1 infection by impairing nuclear entry and subsequent integration. This restriction correlated with the ability of POM987 to bind the assembled HIV-1 capsid core. There is general agreement that HIV-1 nuclear import depends on interactions between the viral capsid and specific nucleoporins or nuclear transport factors (7–12). Capsid mutations such as N74D, which disrupt CPSF6 binding and alter nucleoporin requirements during infection, have been particularly useful for defining these pathways. Cytoplasmic CPSF6 can restrict HIV-1 by binding and sequestering the viral capsid, thereby preventing productive nuclear entry (14).

Like CPSF6, the nuclear pore membrane protein 121 (POM121) is an FG-repeat-containing protein (43, 45, 50). Specifically, POM121C contains a cluster of C-terminal FG repeats with a highly charged alpha-helical motif at its very C-terminus. POM121 is a component of the nuclear pore complex and is located at the inner nuclear membrane of the interphase nucleus where it plays an important role in nuclear pore assembly during interphase (51, 52); POM121 contributes to the nuclear transport of small cellular and viral cargo proteins through nuclear pores by means of a nuclear localization signal that interacts with the classical import receptors importin α/β. We previously reported that N-terminally truncated POM121C (POM987) inhibits HIV-1 replication (46). However, the mechanism underlying this inhibition remained unclear. We previously showed that N-terminally truncated POM121C did not bind to CA in immunoprecipitation studies, had no obvious effect on viral cDNA synthesis, but reduced 2-LTR formation, and inhibited integration of proviral DNA. We now report that POM987 does indeed bind to the assembled capsid, suggesting that POM987 recognizes features of the assembled capsid lattice rather than monomeric or detergent-disrupted CA. Deletion of the highly basic C-terminal alpha-helical motif did not affect protein expression or stability but rendered the resulting POM973 variant incapable of efficiently binding the capsid or inhibiting HIV-1 infection. Although POM987 contains an FG-repeat-rich region, POM973 also retains FG repeats but does not efficiently bind incoming HIV-1 capsids or restrict infection (Fig. 8; lanes 2 and 4). Therefore, FG repeats alone are not sufficient to explain the observed interaction with the viral capsid. Comparison of restrictive and nonrestrictive POM121A isoforms (Fig. 3) may provide further insight into the molecular determinants of POM121–capsid interactions. However, the specific differences among POM121A isoforms and between POM121A and POM121C in virus–host interactions, as well as their structural and functional roles within the NPC, remain unclear. Further studies will be required to address these issues.

A key finding of our study is the identification of viral escape mutants from cells stably expressing POM987. As expected, NL4-3 WT was unable to replicate in cells stably expressing POM987, whereas the expression of POM973 did not restrict HIV-1 replication (Fig. 4A). This phenomenon was not cell-type dependent but was observed in CEM-SS, Jurkat, and MT4 cells (Fig. 5). Interestingly, when parental CEM-SS cells were infected with NL4-3 WT and passaged in the presence of increasing amounts of cells stably expressing POM987, we observed virus replication in cultures of 100% CEM 987 cells starting with the 7th passage (Fig. 4B). We cloned and sequenced virus from these cultures and identified mutations in CA. Introducing these mutations back into NL4-3 WT identified two mutations in the capsid region (R132G and G208R) that, when introduced individually, did not abolish sensitivity to POM987 (Fig. 4C and D). However, when introduced together, the resulting virus was capable of replication in CEM 987 cells (Fig. 4E). Interestingly, the G208R single mutant showed reduced infectivity in POM973-expressing cells during spreading infection (Fig. 4D), although POM973 did not restrict WT HIV-1. The mechanism underlying this G208R-specific phenotype remains unclear, but it may reflect subtle changes in capsid structure, stability, or interaction dynamics that alter sensitivity to POM973. Further studies will be required to define the relevant POM121–capsid interaction interface.

An important feature of the escape phenotype is that escape from POM987-mediated restriction required two CA mutations. The two CA residues involved in the escape phenotype, R132 and G208, are located at structurally distinct positions in the HIV-1 capsid core (Fig. 9), and it remains unclear what interactions or functions these two amino acids perform within the HIV-1 core. R132 is located near the apical CA-NTD site targeted by benzimidazole-based capsid inhibitors (53). Early studies identified the CAP-1 site as the primary binding site for these inhibitors, and resistant variants frequently harbored the G208R substitution together with additional changes near the CAP-1-binding site (54). Indeed, R132 directly participates in inhibitor binding (55). G208R, on the other hand, could alter capsid assembly, morphology, or stability by affecting hexamer–hexamer and hexamer–pentamer trimer interfaces. At these interfaces, substitution of G208 with arginine in adjacent CA subunits could introduce clustered positive charges and potentially destabilize the trimer interface, particularly at the tight pentameric junction. (Fig. 9). It is worth noting that R132G and G208R are reciprocal amino acid changes that preserve the combined net charge at these two positions but may induce structural changes affecting the interaction with POM987.

It is also notable that resistance to CPSF6-mediated restriction can be achieved by a single capsid mutation, such as N74D (14), whereas escape from POM987-mediated restriction required the combination of two capsid mutations, R132G and G208R. Similarly, the antiviral activity of the capsid inhibitor lenacapavir can be reduced by single capsid substitutions, including M66I (36, 56). These comparisons suggest that POM987 targets a capsid-dependent process that may be difficult for HIV-1 to bypass through a single amino acid change. A limitation of the present study is that the experimental system relies largely on overexpression of truncated POM121C fragments. Although expression of these fragments did not measurably affect cell viability under our experimental conditions, the use of overexpressed, truncated proteins does not fully recapitulate the physiological context of endogenous POM121C. In addition, how endogenous full-length POM121C contributes to HIV-1 nuclear entry in physiologically relevant infection settings remains unclear. The potential contribution of POM121A isoforms to HIV-1 capsid interactions and nuclear entry also remains to be defined, particularly because isoform-specific structural and functional differences within the NPC are not fully understood. Future studies will be needed to determine whether specific POM121C domains, POM121A isoforms, or related capsid–nucleoporin interactions can be targeted in a more physiological and therapeutically relevant manner. Taken together, our findings support the broader concept that HIV-1 nuclear entry depends on vulnerable capsid–nucleoporin interactions that may be exploited for antiviral strategies.

## MATERIALS AND METHODS

### Cells

HEK293T and HeLa cells were maintained in Dulbecco’s Modified Eagle’s Medium (DMEM) with 4.5 g/L D-glucose (ThermoFisher Scientific, Waltham, MA) supplemented with 10% heat-inactivated fetal bovine serum (Phoenix Scientific, Laguna Niguel, CA), 100 U/mL penicillin, and 100 µg/mL streptomycin in a 37°C and 5.0% CO_2_ environment. CEM-SS, Jurkat, and MT4 cells were maintained in Roswell Park Memorial Institute (RPMI) 1640 medium supplemented with 10% heat-inactivated fetal bovine serum, 100 U/mL penicillin, and 100 µg/mL streptomycin in a 37°C and 5.0% CO_2_ environment.

### Plasmids and viral vectors

Full-length POM121C (2–987) (NCBI RefSeq: NM_001099415.3), C-terminal domain of POM121A isoform 1 (614–999) (RefSeq: NM_001257190.3), C-terminal domain of POM121A isoform 2 (614–984) (RefSeq: NM_172020.5), and N-terminal domain of CPSF6 (2–321) (RefSeq: NM_007007.3) were amplified by RT-PCR using total RNA isolated from CEM-SS cells. Primer sequences are provided in Table 1. RT-PCR was performed with the PrimeScript II high-fidelity one-step RT-PCR kit (TaKaRa Bio, Shiga, Japan). The amplified RT-PCR products were cloned into the pCR-Blunt II-TOPO vector using Zero Blunt TOPO PCR Cloning Kits (Invitrogen; Thermo Fisher Scientific, Waltham, MA) and verified by sequencing. N-terminally Flag-tagged constructs were created by PCR amplification and cloned into the p3xFlag-CMV-7.1 vector. To generate lentiviral vectors expressing POM121C mutants or CPSF6, Flag-tagged cDNAs were inserted into the pCDH-EF1α-MCS-IRES-Puro vector (System Biosciences, Palo Alto, CA, Cat# CD532A-2). HIV-1 infectious molecular clones pNL4-3 (57) and pNL4-3.Luc.R-E- (HRP-3418) pNL4-3-luc (58) have been reported previously. For construction of capsid (CA) mutants, we first subcloned the NL4-3 gag region between the unique BssHII and AgeI sites into the pCR-Blunt II-TOPO vector. CA mutants were generated by site-directed mutagenesis, and CA-mutated DNA fragments were re-inserted into pNL4-3 or pNL4-3-luc using the unique BssHII and AgeI sites of pNL4-3 and pNL4-3-luc. The resultant plasmids were referred to as pNL4-3/G, pNL4-3/R, pNL4-3/GR or pNL4-3/G-luc, pNL4-3/R-luc, pNL4-3/GR-luc. We also created the CPSF6 escape mutant pNL4-3/N74D-luc using a similar approach. For producing VSV-G pseudotyped virus stocks, pCMV-VSV-G was cotransfected with the appropriate HIV-1 molecular clone.

**TABLE 1:** Primers used for construction of expression vectors

Construction of expression vectors	Forward primer	Reverse primer
pCR-Blunt II-TOPO POM121C (2–987)	GTGTGTAGCCCAGTGACTGT	CTACTTTTTGCGGGTGTGCTG
p3xFlag-POM121C (2–987)	AAAGCGGCCGCAGTGTGTAGCCCAGTGACTGTGAG	AAAAGATCTCTACTTTTTGCGGGTGTGCTG
p3xFlag-POM121C (2–612)	AAAGCGGCCGCAGTGTGTAGCCCAGTGACTGTGAG	TTTAGATCTCTAGGCCGGGGTGAAGCTG
p3xFlag-POM121C (614–987)	TTAAGCTTGGCTCCATATTCCAGTTTGG	AAGAATTCCTACTTTTTGCGGGTGTGCT
p3xFlag-POM121C (614–973)	TTAAGCTTGGCTCCATATTCCAGTTTGG	AAGAATTCCTAAGCCCCTGGGGTCTTGGATC
p3xFlag-POM121 isoform 1 (614–999)	TTAAGCTTGGCTCCATATTCCAGTTTGG	AAGAATTCTCACTCCTTCCTGGTGGGAAAGG
p3xFlag-POM121 isoform 2 (614–984)	TTAAGCTTGGCTCCATATTCCAGTTTGG	AAGAATTCCTACTTTTTGCGGGTGTGCT
pCDH-EF1-3xFlag-POM121C (2–612)-IRES-puro	ACTCTAGAGCCACCATGGACTACAAAGA	TTTAGATCTCTAGGCCGGGGTGAAGCTG
pCDH-EF1-3xFlag-POM121C (614–987)-IRES-puro	ACTCTAGAGCCACCATGGACTACAAAGA	AAGAATTCCTACTTTTTGCGGGTGTGCT
pCDH-EF1-3xFlag-POM121C (614–973)-IRES-puro	ACTCTAGAGCCACCATGGACTACAAAGA	AAGAATTCCTAAGCCCCTGGGGTCTTGGATC
pCDH-EF1-3xFlag-POM121 isoform 1 (614–999)-IRES-puro	ACTCTAGAGCCACCATGGACTACAAAGA	AAGAATTCTCACTCCTTCCTGGTGGGAAAGG
pCDH-EF1-3xFlag-POM121 isoform 2 (614–984)-IRES-puro	ACTCTAGAGCCACCATGGACTACAAAGA	AAGAATTCCTACTTTTTGCGGGTGTGCT
p3xFlag-CPSF6 (2–321)	TTAAGCTTGCGGACGGCGTGGACC	AAGAATTCCTAGCCTGGAGGTGGAGGTGGTCC
pCDH-EF1-3xFlag-CPSF6 (2–321)-IRES-puro	ACTCTAGAGCCACCATGGACTACAAAGA	AAGAATTCCTAGCCTGGAGGTGGAGGTGGTCC
pCR-Blunt II-TOPO NL43 637–3883	GGCGCCCGAACAGGGACTT	GCCCCATCTACATAGAAAGTT
pCR-Blunt II-TOPO NL43 637–3883 CA R132G	GGAGAAATCTATAAAGGATGGATAATCCTGGG	CCCAGGATTATCCATCCTTTATAGATTTCTCC
pCR-Blunt II-TOPO NL43 637–3883 CA G208R	TAAAAGCATTGGGACCTAGGGCGACACTAGAAG	CTTCTAGTGTCGCCCTAGGTCCCAATGCTTTTA
pCR-Blunt II-TOPO NL43 637–3883 CA N74D	TTAAAAGAGACCATCGATGAGGAAGCTGCAGAA	TTCTGCAGCTTCCTCATCGATGGTCTCTTTTAA

### Antibodies

FLAG fusion proteins were detected using a mouse monoclonal anti-FLAG M2-Peroxidase (HRP)-conjugated antibody (Millipore Sigma, St. Louis, MO; Cat# A8592). Tubulin was detected using a mouse monoclonal antibody (Millipore Sigma, St. Louis, MO; Cat# T9026), the nuclear marker Sp1 was identified using a rabbit polyclonal antibody (Santa Cruz Biotechnology Inc., Dallas, TX; Cat#sc-59), Endogenous POM121 was identified using a rabbit polyclonal antibody (GeneTex, Inc., Irvine, CA; Cat. No. GTX102128), LaminB1 was identified using a rabbit polyclonal antibody (Abcam Inc., Cambridge, MA; Cat# ab16048), and HIV-1 CA was identified using pooled HIV Ig (NIH Research and Reference Reagent Program; Cat# 3957).

### Preparation of virus stocks

Virus stocks were prepared by transfecting HEK293T cells (in a 25 cm^2^ flask) with 5 µg of pNL4-3 proviral DNA using TransIT-LT1 Transfection Reagent (Mirus Bio, Madison, WI) according to the manufacturer’s instructions. To produce VSV-g-pseudotyped reporter virus stocks, 0.5 µg of pCMV-VSV-G vector DNA was cotransfected with 4.5 µg of pNL4-3-luc DNA. After 48 h, virus-containing culture supernatants were harvested and precleared by low-speed centrifugation (5 min, 1,500 rpm). The supernatants were then filtered through a 0.45 µm syringe filter. Total amounts of virus in the supernatants were quantified by measuring the virus-associated reverse transcriptase (RT) using a ^32^P-based assay as described below.

### Quantitation of extracellular virus by RT assay

Virus-containing culture supernatants were quantified using a ^32^P-based RT assay as described (59). Briefly, 5 µL of virus-containing culture supernatant was incubated with 30 µL of an RT reaction cocktail containing poly(A) (5 µg/mL), oligo(dT) [oligo(dT)_12–18_; 0.16 µg/mL] in 50 mM Tris, pH 7.8, 7.5 mM KCl, 2 mM dithiothreitol, 5 mM MgCl_2_, 0.05% NP-40, and 1 µCi/mL cocktail of [^32^P]dTTP (800 Ci/mmol). Following a 90-min incubation at 37°C, 5 µL of the reaction mixture was spotted onto DEAE ion exchange paper (Whatman) and washed three times in 2× SSC (0.3 M NaCl and 0.03 M sodium citrate) to remove unincorporated [^32^P] dTTP. Spots were exposed to a Fuji imaging plate for 2–24 h, and levels of bound ^32^P were measured using a Typhoon FLA 9500 (GE Healthcare, Chicago, IL) phosphorimager. The quantitation of the data was done using FujiFilm Multi Gauge software.

### Viral infectivity assay in HEK293T cells

HEK293T cells (1 × 10^6^ cells) were plated in a 6-well plate and transfected the next day with increasing amounts of p3xFlag-POM121C, p3xFlag-POM612, p3xFlag-POM987, or p3xFlag-POM973 (0, 0.1, 0.2, 0.5, and 1.0 µg), adjusted to 2 µg total DNA with an empty vector. At 24 h post-transfection, cells were re-seeded in a 12-well plate at 2 × 10^5^ cells per well, and the next day infected with the VSV-G-pseudotyped HIV-1 reporter virus. After 24 h, the culture medium was aspirated, and cells were lysed in the wells with 300 µL of lysis buffer (25 mM Tris-HCl pH 7.8, 8.0 mM MgCl_2_, 1.0 mM DTT, 1.0% Triton X-100, 15% glycerol). Luciferase activity was determined by combining 30 µL of each lysate with 30 µL of luciferase substrate (Steady-Glo; Promega Corp., Madison, WI). The light emission was measured using a GloMax microplate reader (Promega Corp., Madison, WI).

### Stable expression of POM121C mutants and single-round HIV-1 infection assay

To generate stable POM121C mutant-expressing cells, a lentiviral vector was used in this study. Lentiviral vector stocks were produced by cotransfection of HEK293T cells with pCDH lentiviral vector and pPACKH1 Packaging Plasmid mix (System Biosciences, Palo Alto, CA) according to the manufacturer’s instructions. CEM-SS, Jurkat, or MT4 cells were transduced with lentiviral vectors expressing POM121C mutants and selected with 2 µg/mL puromycin. Cells were plated in a 24-well plate (5 × 10^5^ cells per well in 1 mL medium) and infected with the VSV-G-pseudotyped HIV-1 reporter virus. After 48 h, the cells were lysed with 200 µL of lysis buffer, and luciferase activity was measured as described above.

### Virus replication and identification of escaped viruses by long-term passaging

CEM-SS cells (2 × 10^5^ cells in a 24-well plate) were exposed to HIV-1 NL4-3 stock, and virus production was monitored by measuring RT activity in the culture supernatants. To obtain viruses that escaped from POM987 suppression, NL4-3 was serially passaged in a co-culture of parental and POM987-expressing cells. We started infection with parental cells and POM987-expressing cells at a ratio of 10:1, and virus in the culture medium was collected every 2–3 days and stored at −80°C. After a cytopathic effect was observed, virus replication was confirmed by measuring RT activity. The virus at the peak of RT activity was used for the next infection, with the same or an increased ratio of parental and POM-expressing cells. Sequential passaging of the viruses finally produced viruses that replicated only in POM-expressing cells. Viral RNA was extracted from the culture supernatants at the peak of RT activity using a QIAamp Viral RNA Mini kit (QIAGEN, Valencia, CA). Reverse transcription was performed with SuperScript III Reverse Transcriptase (Invitrogen; Thermo Fisher Scientific, Waltham, MA) using the NL43 3883R primer (GCCCCATCTA CATAGAAAGT T) or the NL43 9173R primer (TGGTGTGTAG TTCTGCCAAT CA). Subsequently, three segmented regions (nucleotide positions 637–3883 [PCR-1], 3780–7627 [PCR-2], and 5949–9173 [PCR-3] of the reference NL4-3) were amplified using PrimeSTAR Max DNA Polymerase2 (TaKaRa Bio). PCR products were initially subjected to direct Sanger sequencing using a primer walking strategy (primer sequences listed in Table 2) to determine consensus-level sequences of the viral population. When mutations or suspected mutations were identified, the corresponding regions were cloned into the pCR-Blunt II-TOPO vector using the Zero Blunt TOPO PCR Cloning Kit (Invitrogen; Thermo Fisher Scientific, Waltham, MA), and at least 15 independent clones were sequenced to confirm the mutations. Sanger sequencing was performed by EZ-Seq (Psomagen, Rockville, MD).

**TABLE 2:** Primers used for identification of escaped viruses

Usage	Primer name		Primer sequence	nt position	
RT	NL43 3883R	Reverse	GCCCCATCTACATAGAAAGTT	3863	3883
RT	NL43 9173R	Reverse	TGGTGTGTAGTTCTGCCAATCA	9152	9173
PCR-1	NL43 637F	Forward	GGCGCCCGAACAGGGACTT	637	655
PCR-1	NL43 3883R	Reverse	GCCCCATCTACATAGAAAGTT	3863	3883
PCR-2	NL43 3780F	Forward	ATTCCTGAGTGGGAGTTTG	3780	3798
PCR-2	NL43 7627R	Reverse	TCCAGGTCTGAAGATCTCG	7609	7627
PCR-3	NL43 5949F	Forward	AAAAGCCTTAGGCATCTCC	5949	5967
PCR-3	NL43 9173R	Reverse	TGGTGTGTAGTTCTGCCAATCA	9152	9173
Sanger sequencing	NL43 637F	Forward	GGCGCCCGAACAGGGACTT	637	655
Sanger sequencing	NL43 975F	Forward	ACAGCTACAACCATCCC	975	991
Sanger sequencing	NL43 991R	Reverse	GGGATGGTTGTAGCTGT	975	991
Sanger sequencing	NL43 1611F	Forward	AAGAATGTATAGCCCTACC	1611	1629
Sanger sequencing	NL43 1629R	Reverse	GGTAGGGCTATACATTCTT	1611	1629
Sanger sequencing	NL43 2314F	Forward	AAGCTCTATTAGATACAGG	2314	2332
Sanger sequencing	NL43 2332R	Reverse	CCTGTATCTAATAGAGCTT	2314	2332
Sanger sequencing	NL43 2860F	Forward	AATCAGTAACAGTACTGG	2860	2877
Sanger sequencing	NL43 3413F	Forward	ACTAACAGAAGTAGTACC	3413	3430
Sanger sequencing	NL43 3780F	Forward	ATTCCTGAGTGGGAGTTTG	3780	3798
Sanger sequencing	NL43 3883R	Reverse	GCCCCATCTACATAGAAAGTT	3863	3883
Sanger sequencing	NL43 4704F	Forward	AAGAAAATTATAGGACAGG	4704	4722
Sanger sequencing	NL43 5326F	Forward	ACACAAGTAGACCCTGAC	5326	5343
Sanger sequencing	NL43 5623R	Reverse	AGCTCTAGTGTCCATTCATTGTATG	5599	5623
Sanger sequencing	NL43 5949F	Forward	AAAAGCCTTAGGCATCTCC	5949	5967
Sanger sequencing	NL43 6582F	Forward	AATTAACCCCACTCTGTG	6582	6599
Sanger sequencing	NL43 7393F	Forward	TCTTTAAGCAATCCTCAG	7293	7310
Sanger sequencing	NL43 7499F	Forward	CAGGAAGTAGGAAAAGCAATGT	7499	7517
Sanger sequencing	NL43 7627R	Reverse	TCCAGGTCTGAAGATCTCG	7609	7627
Sanger sequencing	NL43 7934F	Forward	AAACAGCTCCAGGCAAGAATCC	7934	7955
Sanger sequencing	NL43 8606F	Forward	AATCTCCTACAGTATTGG	8606	8623

### Immunoblot analysis

For preparation of cell extracts, cells were washed once with PBS, suspended in PBS (100 µL/10^6^ cells), and mixed with an equal volume of 2× sample buffer (4% SDS, 125 mM Tris, pH 6.8, 10% 2-mercaptoethanol, 10% glycerol, and 0.001% bromophenol blue). Samples were heated at 95°C with occasional vortexing until samples were completely dissolved. Samples were subjected to SDS-PAGE and transferred to polyvinylidene difluoride (PVDF) membranes. Membranes were incubated for 30 min with non-fat dry milk (5% solution in 1× TNT buffer [10 mM Tris pH 7.4, 150 mM NaCl, 0.3% Tween-20]). Membranes were washed once with TNT-N buffer (TNT + 0.05% IGEPAL CA-630), followed by one wash in TNT buffer (2 min each). Membranes were then reacted for 1 h or overnight with primary antibodies as described in the text. Unbound antibodies were removed by washing membranes in TNT-N and TNT (one wash each, 5 min). Membranes were then incubated with horseradish peroxidase-conjugated secondary antibodies (GE Healthcare, Piscataway, NJ), and proteins were visualized by Clarity Western ECL substrate (Bio-Rad, Hercules, CA; Cat# 170-5061) or Immobilon Western chemiluminescent HRP substrate (Millipore Sigma, St. Louis, MO; Cat# WBKLS0500). Images were acquired using a ChemiDoc imaging system (Bio-Rad, Hercules, CA) and were quantified using Image Lab (v 6.0) software.

### Quantification of viral cDNA

Prior to infection, virus stock was treated with 50 U/mL of RNase-free DNase I in the presence of 10 mM MgCl_2_ for 30 min at 37°C. CEM-SS cells (5 × 10^5^ cells in a 24-well plate) were infected with RT activity-normalized VSV-G-pseudotyped NL4-3luc virus for 24 h. Total cellular DNA was extracted using DNeasy Blood & Tissue Kit (QIAGEN, Valencia, CA). Virus was heat inactivated by incubation at 65°C for 30 min and was used as a negative control.

Quantitative PCR assays were performed with 200 ng of total DNA using TaqMan Fast Advanced Master Mix for qPCR (ThermoFisher Scientific, Waltham, MA, Cat# 4444964), and the reactions were run using a CFX96 Touch real-time PCR detection system (Bio-Rad, Hercules, CA). Primers and probe for qPCR were as follows: pol-F (5′-ATTCCTGAGT GGGAGTTTG-3′) (nt 3780–3798), pol-R (5′-AACTTTCTAT GTAGATGGGG C-3′) (nt 3863–3883) and pol-probe (5′- FAM-CAATACCCCT CCCTTAGTGA AGTTATGGTA C-TAMRA-3′) (nt 3800–3830) were used for amplification and detection of the pol region of the HIV-1 NL4-3 late reverse transcription product; 2LTR-S (5′-CCCTCAGACC CTTTTAGTCA GTG-3′) (nt 9668–9690), 2LTR-AS (5′-TGGTGTGTAG TTCTGCCAAT CA-3′) (nt 77-98), and 2LTR-probe (5′-FAM-TGTGGATCTA CCACACACAA GGCTACTTCC-TAMRA-3′) (nt 46–75) were used for the 2-LTR circle from. Quantification of integrated viral DNA was performed using a modified Alu-PCR method as previously described (46, 60). Because lentiviral vectors were introduced into the cells in this study, amplification targeting the LTR region resulted in high background signals. Therefore, the first PCR was performed using PrimeSTAR GXL DNA polymerase (TaKaRa Bio) with Alu-HIV (TCCCAGCTAC TCGGGAGGCT GAGG) and NL4-3 5623R (AGCTCTAGTG TCCATTCATT GTATG) primers. The PCR conditions were 22 cycles of denaturation (98°C for 10 s), annealing (60°C for 5 s), and extension (68°C for 10 min). The first PCR products were diluted 10-fold and used as templates for qPCR. The qPCR was performed targeting the pol region, as described above for late reverse transcription products. To normalize the amount of DNA in each qPCR assay, the following primers and probe were used to quantify the cellular β-globin cDNA: huBG/F (5′-CAAGAAAGTG CTCGGTGCCT T-3′), huBG/R (5′-CCTGAAGTTC TCAGGATCCA CG-3′), and b-globin/probe (HEX) (5′-HEX-ACACTGAGT/ZEN/G AGCTGCACTG TGACAAGCTG-Iowa BlackFQ-3′). The ratios of each viral cDNA to β-globin DNA were calculated, and the CEM-SS value was defined as 100%. All primer and probe sequences used for qPCR analysis are listed in Table 3.

**TABLE 3:** qPCR primer probe list

Usage	Primer name	Sequence
Late RT (pol region)	pol-F (3780–3798)	ATTCCTGAGTGGGAGTTTG
Late RT (pol region)	pol-R (3863–3883)	GCCCCATCTACATAGAAAGTT
Late RT (pol region)	pol-probe (FAM) (3800–3830)	5′-FAM-CAATACCCCTCCCTTAGTGAAGTTATGGTAC-TAMRA-3′
2LTR	2LTR-S (9668–9690)	CCCTCAGACCCTTTTAGTCAGTG
2LTR	2LTR-AS (77–98)	TGGTGTGTAGTTCTGCCAATCA
2LTR	2LTR-FAM (probe) (46–75)	5′-FAM-TGTGGATCTACCACACACAAGGCTACTTCC-TAMRA −3′
Integrated form 1st PCR	Alu-HIV	TCCCAGCTACTCGGGAGGCTGAGG
Integrated form 1st PCR	NL43 5623R	AGCTCTAGTGTCCATTCATTGTATG
Internal control (b-globin)	huBG/F	CAAGAAAGTGCTCGGTGCCTT
Internal control (b-globin)	huBG/R	CCTGAAGTTCTCAGGATCCACG
Internal control (b-globin)	b-globin/probe (HEX)	5′-HEX-ACACTGAGT/ZEN/GAGCTGCACTGTGACAAGCTG-Iowa BlackFQ-3′

### Subcellular fractionation to detect HIV-1 capsid

To inhibit newly synthesized viral proteins, an integrase inhibitor (100 nM Raltegravir) was added to the culture medium. CEM-SS cells (2 × 10^6^ cells in a 12-well plate) were exposed to RT activity-normalized NL4-3 wild-type or NL4-3 CA mutant at 4°C for 15 min. Cells were further incubated at 37°C for the indicated times, then collected and washed twice with cold PBS. Nuclear-cytoplasmic protein extraction was conducted using the NE-PER Nuclear and Cytoplasmic Extraction Reagents kit (ThermoFisher Scientific, Waltham, MA, Cat# 78835) according to the manufacturer’s instructions. Cytoplasmic and nuclear extracts were analyzed by immunoblot.

### Protein expression and purification for CA tube assembly

pET-11a vectors were used to express the HIV-1 capsid protein in *E. coli*. Point mutations A14C and E45C were introduced using the QuikChange II site-directed mutagenesis kit (Agilent, Santa Clara, CA) according to the manufacturer’s instructions. All proteins were expressed in *Escherichia coli* one-shot BL21starTM (DE3) cells (Invitrogen; Thermo Fisher Scientific, Waltham, MA). Briefly, LB medium was inoculated with overnight cultures, which were grown at 30°C until mid-log-phase (A600, 0.6–0.8). Protein expression was induced with 1 mM isopropyl-β-d-1-thiogalactopyranoside (IPTG) overnight at 18°C in a shaker incubator. Cells were harvested by centrifugation at 5,000 × *g* for 10 min at 4°C, and the pellets were stored at −80°C until purification. Purification of capsid was carried out as follows. Pellets from 2 L of bacteria were lysed by sonication (Qsonica microtip: 4420; A = 45; 2 min; 2 s on; 2 s off for 12 cycles), in 40 mL of lysis buffer (50 mM Tris pH 8.0, 50 mM NaCl, 100 mM 2-mercaptoethanol, and Complete EDTA-free protease inhibitor tablets). Cell debris was removed by centrifugation at 40,000 × *g* for 20 min at 4°C. Proteins from the supernatant were precipitated by incubation with 1/3 of the volume of saturated ammonium sulfate containing 100 mM 2-mercaptoethanol for 20 min at 4°C and centrifugation at 8,000 × *g* for 20 min at 4°C. Precipitated proteins were resuspended in 30 mL of buffer A (25 mM MES, pH 6.5, 100 mM 2-mercaptoethanol) and sonicated 2–3 times (Qsonica microtip: 4420; A = 45; 2 min; 1 s on; 2 s off). The sample was dialyzed three times in buffer A (2 h, overnight, 2 h). The sample was sonicated and diluted in 500 mL of buffer A and was chromatographed sequentially on a 5 mL HiTrapTM Q HP column and on a 5 mL HiTrapTM SP FF column (GE Healthcare), both preequilibrated with buffer A. The capsid protein was eluted from the HiTrapTM SP FF column using a linear gradient from 0 to 2 M of NaCl. Absorbance at 280 nm was checked to take the eluted fraction that had higher protein levels. Pooled fractions were dialyzed three times (2 h, overnight, 2 h) in storage buffer (25 mM MES, 2 M NaCl, and 20 mM 2-mercaptoethanol). The sample was concentrated using Centricon filters to a concentration of 20 mg/mL and stored at −80°C.

### Assembly of stabilized HIV-1 capsid tubes

An amount of 1 mL of monomeric capsid (3 mg/mL or 1 mg/mL) was dialyzed in SnakeSkin dialysis tubing 10,000 MWCO (Thermo Fisher Scientific) against buffer 1 (50 mM Tris pH 8.0, 1 M NaCl, 100 mM 2-mercaptoethanol) at 4°C for 8 h. Subsequently, the protein was dialyzed against the same buffer without the reducing agent 2-mercaptoethanol (buffer 2: 50 mM Tris pH 8.0, 1 M NaCl) at 4°C for 8 h. The absence of 2-mercaptoethanol in the second dialysis step allows the formation of disulfide bonds between Cysteine 14 and 43 inter-capsid monomers in the hexamer. Finally, the protein was dialyzed against buffer 3 (20 mM Tris pH 8.0, 40 mM NaCl) at 4°C for 8 h to allow capsid assembly. Assembled complexes were stored at 4°C for up to 1 month.

### Capsid binding assay protocol

Human HEK293T cells were transfected for 24 h with a plasmid expressing Flag-tagged POM987 or POM973. Cell media was completely removed, and cells were lysed in 300 μL of capsid binding buffer (CBB: 10 mM Tris pH 8.0, 1.5 mM MgCl_2_, and 10 mM KCl) by scraping off the plate. Cells were rotated at 4°C for 15 min and then centrifuged to remove cellular debris (21,000 × *g*, 15 min, 4°C). Cell lysates were incubated with stabilized HIV-1 capsid tubes for 1 h at 25°C. Subsequently, stabilized HIV-1 capsid tubes were washed by pelleting the complexes at 21,000 × *g* for 2 min. Pellets were washed by resuspension in PBS. Pellets were resuspended in 1× Laemmli buffer and analyzed by immunoblotting using anti-Flag and anti-p24 antibodies.

### Statistical analysis

The average values of all data presented with error bars reflect the standard error of the mean (SEM). Statistical significance was determined using one-way ANOVA with Dunnett’s *post hoc* test. The null hypothesis was rejected if the *P* value was less than the significance levels, i.e., *P ≤ 0.05; **P ≤ 0.01; ***P ≤ 0.001; n.s.: non-significant.

## Data Availability

The HIV-1 capsid structure used for Fig. 9 is available from the RCSB Protein Data Bank under accession code 8G6K (https://www.rcsb.org/structure/8G6K). No custom software or new publicly archived data sets were generated in this study. Additional raw data supporting the findings of this study are available from the corresponding author upon request.
